# Does biofilm formation have different pathways in *Staphylococcus aureus?*

**DOI:** 10.22038/ijbms.2019.34888.8281

**Published:** 2019-10

**Authors:** Ali Shivaee, Behrooz Sadeghi Kalani, Malihe Talebi, Davood Darban-Sarokhalil

**Affiliations:** 1 Microbial Biotechnology Research Center, Department of Microbiology, School of Medicine, Iran University of Medical Sciences, Tehran, Iran

**Keywords:** Biofilm, MRSA, MSSA, PIA, Staphylococcus aureus

## Abstract

**Objective(s):**

Biofilm formation is one of the most important factors in the development of infections caused by *Staphylococcus aureus.* In this study, the expression levels of genes responsible for biofilm formation were studied in methicillin sensitive and methicillin resistant *S. aureus.*

**Materials and Methods:**

A total of 100 meticillin-resistant *s.aureus* (MRSA) and meticillin-sensetive s.aureus (MSSA) isolates were studied. Bacterial biofilm formation was evaluated phenotypically using microtiter plate method. Real-time PCR tests were conducted to determine the expression levels of genes involved in biofilm formation.

**Results:**

Quantitative biofilm formation test was repeated three times for each specimen. The prevalence of weak, medium, and strong biofilm producers were 16%, 49%, and 35%, respectively. In MSSA isolates, expression levels of *ica* genes increased compared to the *fnbA, fnbB, clfA* and *clfB* genes. These results were different in MRSA isolates, and *ica* genes showed a decreased gene expression levels compared to the aforementioned genes.

**Conclusion:**

Considering the results of this study, *clf* genes probably contribute to the same extent in both MRSA and MSSA isolates, and there is probably no significant difference in the role of these genes in these isolates. In addition, the results of this study indicated that MRSA may not use the conventional route for biofilm formation and may use independent pathways through Polysaccharide intercellular adhesion (PIA).

## Introduction

*Staphylococcus aureus* is a Gram-positive facultative anaerobic cocci. This bacterium is the most important clinical species among Staphylococci group (1). *S. aureus* was primarily isolated by a Scottish surgeon, Alexander Ogston, from an infectious abscess specimen in 1880 (2). This microorganism is considered as one of the most successful pathogenic bacteria successively colonizing human and animal skin and mucus. This microorganism is also considered as a potential human pathogen capable of causing infection in different body sites including skin, soft tissue, respiratory routes, skeleton, and the joints. This microorganism is a leading cause of nosocomial and community-acquired infections causing a variety of lethal human infections including endocarditis, osteomyelitis, pneumonia, and urinary tract infection (3-8). *S. aureus* is currently categorized in two groups of methicillin-sensitive *S. aureus *(MSSA) and methicillin-resistant *S. aureus* (MRSA). Methicillin-sensitive species were commonly isolated prior to the emergence of MRSAs (8). Methicillin-resistant bacteria are also resistant to other antibiotics (9). In 2007, studies showed that more than 65% of nosocomial infections were caused by biofilm-forming microorganisms (10). *S. aureus* is also a common pathogen associated with biofilm-related infections and a contaminant of medical devices, causing infection in a large number of patients annually. *S. aureus* has a high prevalence on human skin and mucus compared with other biofilm-forming pathogens (11). In MRSA and MSSA isolates, biofilm formation is considered as a virulence factor leading to the resistance to antibiotics and the toleration of harsh environmental conditions. Infections caused by this microorganism include endocarditis, septicemia, osteomyelitis, catheter-related urinary tract infection, and ventilator-related pneumonia (12, 13).

Biofilm formation on surfaces such as biological materials is possibly a major factor in the spread of the infection. Biofilm formation facilitates the aggregation and adherence of microorganism to solid surfaces (14). Adherence to various surfaces is a fundamental step in biofilm formation and bacterial aggregation. This adherence is facilitated by bacterial surface proteins called ‘‘ The Microbial Surface Components Recognizing Adhesive Matrix Molecules’’ (MSCRAMMs) (15). In fact, bacteria are able to produce biofilms through the formation of polysaccharide matrix. Polysaccharide intercellular adhesion (PIA) encoded by *ica* operon is required for biofilm formation in *S. aureus* (16). PIA is in fact a polysaccharide encoded by *ica*ADBC operon and is formed by partially deacetylated β (1→6) glycose aminyl glycan bonds. This polysaccharide can surround the cell, protecting it against human immune system and antibiotics (17). In 2001, Cucarella, *et al.* reported PIA-independent biofilm formation in *S. aureus* isolated from sheep breast abscess. In these isolates, biofilm formation was mediated by biofilm-associated proteins (Bap) (18). *ica*-independent biofilm formation was confirmed in human *S. aureus *isolates (UAMS-1) *in vivo* and *in vitro* (19). Studies suggest that acquiring resistance to methicillin can lead to the suppression of PIA and surface protein-dependent biofilm formation (20). 

This study aimed to investigate the expression levels of genes associated with biofilm formation in MRSA and MSSA isolated from clinical specimens in different cities of Iran. Understanding the expression levels of these genes help us to understand genetic mechanisms used by MRSA and MSSA isolates and to compare genetic pathways used by these two isolates.

## Materials and Methods

***Isolation of S. aureus***

In this study, a total of 100 MRSA and MSSA isolates (isolated from burn wounds) were investigated. Bacteria were previously identified in the Microbiology Lab of Iran University of Medical Sciences by biochemical and molecular tests. 

**Figure 1:**
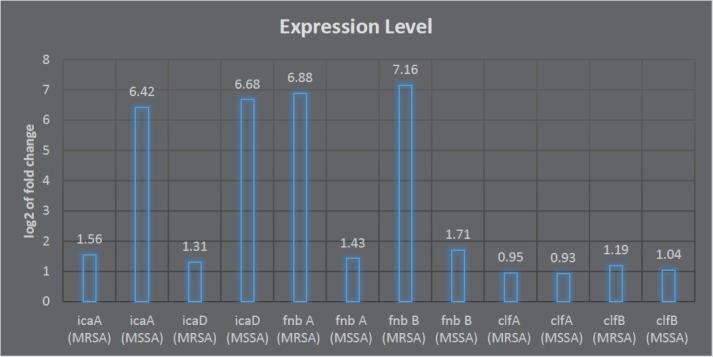
Results of changes in expression level of the studied genes after biofilm induction compared to the control sample in MRSA and MSSA isolates

MRSA: meticillin-resistant *s.aureus*; MSSA: meticillin-sensetive *s.aureus*

**Figure 2:**
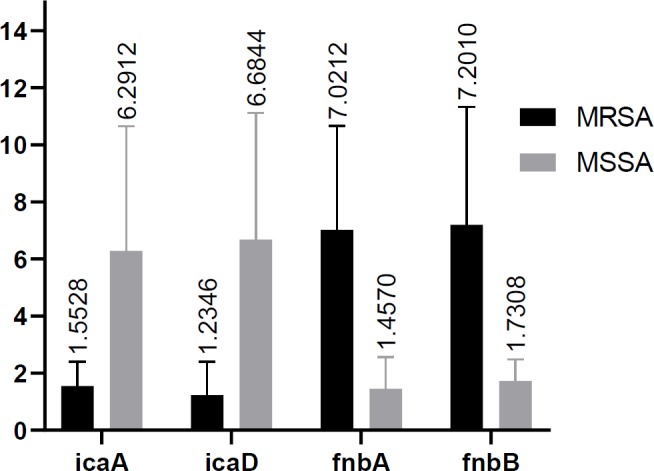
Statistical analysis of *ica* and *fnb* genes among MRSA and MSSA isolates

MRSA: meticillin-resistant *s.aureus*; MSSA: meticillin-sensetive *s.aureus*

**Figure 3:**
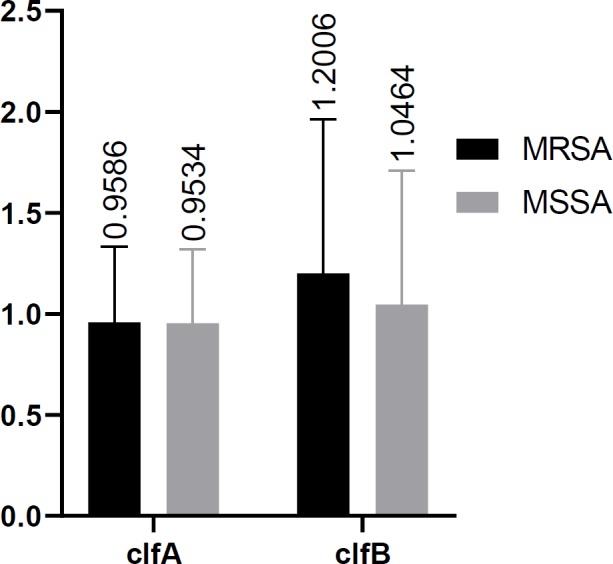
Statistical analysis of *clf *gene between MRSA and MSSA isolates

MRSA: meticillin-resistant *s.aureus*; MSSA: meticillin-sensetive *s.aureus*

**Figure 4:**
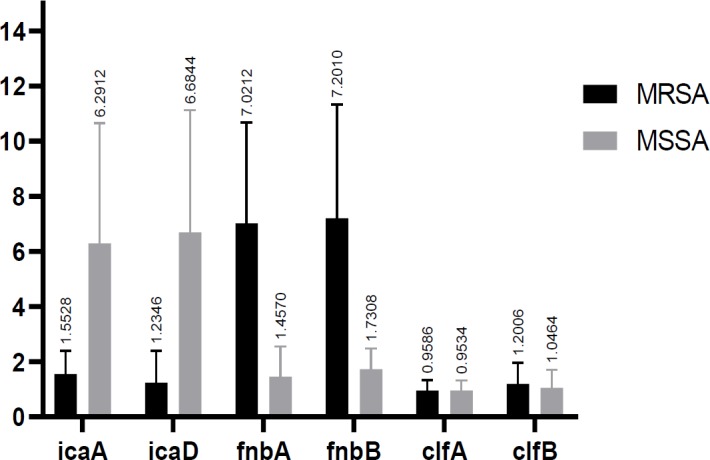
Statistical comparison of the studied genes among MRSA and MSSA isolates

MRSA: meticillin-resistant *s.aureus*; MSSA: meticillin-sensetive *s.aureus*

**Table 1:** Primers used in this study for qPCR

**Reference**	**Annealing temperature**	**Sequence**	**Gene**	**NO**
(35)	59	F: GAGGTAAAGCCAACGCACTC R: CCTGTAACCGCACCAAGTTT	*icaA*	1
(35)	56	F: ACCCAACGCTAAAATCATCG R: GCGAAAATGCCCATAGTTTC	*icaD*	2
(36)	55	F: TGAAGGTGGTTATGTTGATG R: CAGTGTATCCTCCAACATGA	*fnbA*	3
(36)	60	F: GTAGAGGAAAGTGGGAGTTCAG R: TGTGTTGATTGTGATGGTTGC	*fnbB*	4
(36)	55	F: TACAAGTGCGCCTAGAATGA R: TTTGACATAACCTGCTTGGT	*clfA*	5
(36)	59	F: GTGTAGATACAGCTTCAGGTCA R: CACTTACTTTACCGCTACTTTC	*clfB*	6
(36)	53	F: AGGTCTTGGAGAAATGAATG R: CAAATGTTTGGTCCGCTT	*gyrB*	7

**Table 2:** Conditions required for real-time PCR reaction

**Cycle**	**Cycle Point**
**Hold @ 95°c, 10 min 0 s**	
**Cycling (40 repeats) **	Step 1 @ 95°c, hold 20 s
	Step 2 @ TM°c(3-6 table), hold 25 s
	Step 3 @ 72°c, hold 20 s, acquiring to Cycling A([Green][1][1])
**Melt (72-99°c) , hold secs on the 1st step, hold 5 secs on next steps, Melt A([Green][1][1])**	

**Table 3:** Results of adhesion formation by microtiter plate method

**Isolates %**				**Test**	
Highly (%)	Moderate (%)	Weak (%)	None (%)	Biofilm formation	
19 (19%)	26 (26 %)	9 (9%)	0(0%)	MRSA	Standard microtiter plate method
16 (16%)	23 (23%)	7 (7%)	0(0%)	MSSA	
35%	49%	15%	0(0%)	Total	

**Table 4:** Statistical analysis of different genes among the isolates

variable	number	Group	Mean	Standard deviation	Test	Results
***IcaA***	50	MRSA	1.5544	.840513	T-test	T=-7.541 df=52.63 P-value < 0.001
	50	MSSA	6.2926	4.36763		
***icaD***	50	MRSA	1.2353	1.16400	Mann-Whitney	Z=-7.671 P-value < 0.001
	50	MSSA	6.6845	4.44121		
***fnbA***	50	MRSA	7.0214	3.65111	Mann-Whitney	Z=-8.009 P-value < 0.001
	50	MSSA	1.4571	1.10468		
***fnbB***	50	MRSA	7.2008	4.13546	Mann-Whitney	Z=-7.46 P-value < 0.001
	50	MSSA	1.7308	.75027		
***clfA***	50	MRSA	.9600	.37710	T-test	T= 0.900 df=98 P-value = 0.929
	50	MSSA	.9533	.36982		
***clfB***	50	MRSA	1.2023	.76198	T-test	T=1.089 df=98 P-value = 0.279
	50	MSSA	1.0467	.66331		

***In vitro biofilm formation assay***

The ability of biofilm formation was investigated in MRSA and MSSA isolates according to the previous studies (21). Briefly, 250 μl of both MRSA and MSSA isolates diluted 1:1000 in tryptic soy broth (TSB), supplemented with 1% glucose were inoculated in 96-well polystyrene microtitre plates and were incubated for 94 hr at 37 ^º^C. After biofilm formation, non-adherent bacterial cells were removed and washed twice with 200 μl of sterile phosphate buffer saline (PBS; pH 7.0). Cell were then stained with 300 μl of crystal violet (2%) after being dried in an inverted position at room temperature under laminar air flow. Then, the stained cells were washed 3 times with distilled water in order to remove the extra stain and 300 μl of ethanol: acetic acid (95:5 v/v) solution was added to each well. Then, 100 μl of this solution was transferred to another 96- well plate and absorbance was measured at 570 nm using Elisa reader (Merck, USA). Culture medium was used as a control. Isolates were classified in three categories: strong (OD_570_≥0.5), medium (OD_570_≥0.2 to <0.5) and weak (OD_570_ 0 to <0.2) biofilm producers (22). Each test was done in triplicates.

***RNA isolation***

In order to induce biofilm formation, isolates from two groups of MRSA and MSSA were inoculated in TSB culture medium containing glucose 1% in 96-well microplate. The plates were then incubated at 37 ^º^C for 18 hr. 

In brief, cells were washed in 6-well polystyrene plates with ddH_2_O three times. Bacterial cells with adherence to each well were disrupted and scraped from the plate surface by sterile micropipette tips and re-suspended in cold sterile distilled water. After centrifugation, pellets were washed with deionized water to remove the planktonic cells. Cells were centrifuged again and the supernatant was discarded. The cells were initially resuspended in tris-EDTA buffer (pH 7.5) containing 15 mg/ml lysozyme (Sigma-Aldrich), and 0.1 mg/ml lysostaphin (Sigma Aldrich). Then, the pellet was subsequently processed using the High pure RNA isolation kit (Roche, Germany) according to the manufacturer’s protocol.

The quality and quantity of RNA was determined by agarose gel electrophoresis and by measuring the absorbance at 260 and 280 nm using a Nanodrop spectrophotometer ND-1000 (Thermo Fisher Scientific, Wilmington, DE, USA). The Purified RNA was immediately converted to cDNA to avoid RNA degradation using first script RT reagent kit (Takara, Japan (according to the manufacturer’s instructions. 

***Primers design for qPCR***

The primers were used in this study are shown in Table1. Annealing temperatures were optimized for each primer pair by melting curve analysis and by post-PCR agarose gel electrophoresis. The identities of all PCR products were confirmed and the amplification efficiency for each primer set was determined by a RT-qPCR assay in order to evaluate the linearity of target amplification.

***Quantitative real-time PCR***

In order to understand the difference on genetic pathways of biofilm formation, 2-step real-time PCR was performed on 6 genes involved in biofilm formation. *gyr*B gene was used as an internal control. The reaction was carried out in a Qiagen ^r ^(roto gene 6000) using the SYBR Green Master Mix (Takara, japan) according to the manufacturer’s protocol. In brief, for each reaction, 2 µl of sample, 0.8 µl of each primer (Forward and Reverse) with the concentration of 10 picomol, 10 µl of SYBR Premix, and 6 µl of distilled water were used. The reaction started with an initial denaturation at 95 ^º^C for 5 min and 40 amplification cycles of 95 ^º^C for 20 sec, TM annealing for 25 sec and 72 ^º^C for 20 sec (Table 2).

***Data analysis***

For each sample, curves were drawn for both the target and endogenous references. Data were then subjected to analysis using the Relative Expression Software Tool (REST) program (Qiagen) and Rotor gene 6000 application.

***Statistical analysis***

Since data on *icaA*, *clfA*, and *clfB* genes were normalized, Independent Samples T-test was performed to compare them among MRSA and MSSA groups. Also, since data on *icaD*, *fnbA*, and *fnbB* genes were not normalized, Mann-Whitney test was performed to compare these genes among MRSA and MSSA groups.

## Results

***Biofilm formation assay***

Quantitative test for biofilm formation was repeated three times for each sample and finally, optical absorbance was read by ELISA reader at 570 nm. Weak, intermediate, and strong biofilm formation were observed in 16 (16%), 49 (49%), and 35 (35%) of the isolates (Table 3), respectively. 

***Real-time PCR***

Values of CT for *icaA* , *icaD* , *fnbA*, *fnbB*, *clfA*, and *clfB* genes were compared with calibrated sample and calculated by Rotor gene. Among 100 studied isolates, 50 MRSA isolates and 50 MSSA isolates, higher expression levels of *ica *were observed in MSSA isolates compared to *fnbB*, *clfA* and *clfB*. These results were different in MRSA isolates and *ica* genes showed lower expression levels compared to other genes. Then, fold change of the studied genes in MRSA and MSSA isolates was calculated. In MSSA isolates, fold changes for *icaA* , *icaD* , *fnbA* , *fnbB* , *clfA* , * clfB* genes were 6.4, 6.6, 1.4, 1.7, 0.9, and 1, respectively (Figure 1). Interpretation of the results of fold change showed a slight difference in expression level of *clf* in MRSA and MSSA isolates. 

***Statistical analysis***

Results showed the significant difference between the expression levels of *icaA*, *icaD, fnbA*, and *fnbB* in the two studied groups (p-value< 0.05). However, no significant difference was observed between the expression levels of *clfA* and *clfB* among two studied groups (p value >0.05) (Table 4). Among MSSA and MRSA groups, *icaA* and *icaD* have the highest expression levels with the mean values of 6.4292 and 6.6869, respectively. While, *fnbA* and *fnbB* have higher expression levels of 7.1648 and 6.8864 compared to MSSA group (Figures 2-4).

## Discussion

*S. aureus* is considered as the most important clinical species in Staphylococci genus (1). It is estimated that roughly 30% of all humans are asymptomatic carriers of this pathogen (2). This bacterium is also a common pathogen in biofilm-associated infections and medical device contaminations. This bacterium has a high prevalence compared to other biofilm-forming bacteria (11). Biofilm formation in MRSA and MSSA isolates is considered as a virulence factor and helps the bacteria to overcome harsh environmental conditions and to become resistant to antibiotics, causing various infections including endocarditis, septicemia, osteomyelitis, catheter-related urinary tract infection, and ventilator-related pneumonia. Moreover, there are different molecules such as collagen, ﬁbronectin and ﬁbrinogen in burn wounds, *S. aureus* by encoding many proteins that specifically interact with human cellular matrix components can colonize burn wounds (12, 13). The ability of biofilm formation on biological materials is considered as a major factor in the spread of the infection (14). In this study, 50 MRSA and 50 MSSA isolates from burn wounds were investigated and biofilm formation was observed among all the isolates. Weak, intermediate, and strong biofilm formation were observed among 16%, 49%, and 35% of isolates, respectively. These results were compatible with the results of the studies of Moghadam *et al.* in 2014. They concluded that among 65 isolates, 97% of MRSA isolates and 70% of MSSA isolates had the ability of biofilm formation (25). Also, in another study by Vasudevan *et al.* in 2010, among 35 studied isolates, 32 (91%) were capable of biofilm formation (26) which was similar to our study. Rohde *et al.* showed similar results in Staphylococci isolated from knee surgery. They reported that all isolates were capable of biofilm formation (27). However, results of our study showed higher biofilm formation compared to the studies conducted by Adilson Oliveira *et al.* in 2010 (46% of isolates showed strong adherence and 35% of isolates showed weak adherence), Kwon *et al.* in 2008, Knobloch *et al.* in 2004, and Fowler *et al.* in 2002 (28-31). These studies show that the prevalence of biofilm formation has increased in recent years which suggests the importance of the studies on biofilm formation pathways and novel therapeutic strategies. PIA secretory proteins and surface proteins encoded by *ica* operon are required in biofilm-producing bacteria. This operon provides the necessary proteins for PIA synthesis in Staphylococci species (16). One of the first reports on the presence of PIA-independent biofilm formation in *S. aureus* was given by Cucarella *et al.* in 2001. They were able to observe biofilm formation by biofilm-associated proteins (Bap) in sheep breast abscess isolates (18). Further studies suggest that acquiring resistance to methicillin can suppress surface protein and PIA-dependent biofilm formation (20). This challenge about PIA-independent biofilm formation shows that biofilm production and propagation has not yet been fully understood which suggest the need for studying different pathways for biofilm formation. The current study aimed to investigate the presence of different biofilm formation pathways in MRSA and MSSA isolates by real-time PCR and biofilm induction methods. This study was conducted on 50 MRSA and 50 SSA isolates. Our results showed the higher expression levels of *ica* gene in MSSA isolates compared to *clfA*, *clfB, fnbA*, and *fnbB*. These results were different in MRSA isolates and *ica* genes showed lowed expression levels compared to other genes. Fold change calculation for *icaA, icaD, fnbA, fnbB, clfA,* and *clfB* genes were 1.4, 1.5, 6.8, 7.1, 1.1, and 1.19, respectively. While, in MSSA isolates, these values were 6.4, 6.6, 1.4, 1.7, and 0.9, respectively. Fold change interpretations and statistical analyses clearly showed that there is a significant relationship between acquiring resistance to methicillin and pathways for biofilm formation. Results showed the increase in using PIA-independent pathway for biofilm formation in MRSA isolates. This was completely different for MSSA isolates which show PIA-dependent biofilm formation. Similar studies in 2010 were conducted by Boles *et al.* for the identification of the genes involved in PIA-independent biofilm formation in *S. aureus*. By inducing mutation in the expressed genes involved in PIA-independent biofilm formation in MRSA, they showed the intense decrease in biofilm formation levels in these isolates (32). In another study by O’neill *et al.* in 2007, biofilm formation in MRSA and MSSA isolates was investigated by the deletion of *ica* locus. They showed the capability of biofilm formation in MRSA isolates, but not in MSSA isolates (33). In 2008, they also showed that biofilm formation in MRSA isolates is affected and reduced by the deletion of *fnbA* and *fnbB* genes; while, this mutation in biofilm formation did not affect MSSA isolates (34). In the study of Shanks *et al.* it was suggested that MRSA isolates use PIA-independent pathways for biofilm formation (35). Also, in another similar study by Dastgheib *et al.* in 2014, it was suggested that MRSA isolates use *fnb* and *clf* genes for PIA-independent biofilm formation in joint fluids (36). However, our study did not show a significant difference among MSSA and MRSA isolates. Fold change comparison and statistical analysis shows that there is no significant difference in using *clf* gene in MRSA and MSSA isolates and it is probably used at the same levels in both MRSA and MSSA isolates. Unfortunately, no specific study has been conducted for these gene; therefore, more studies are required to better understand the role of this gene in biofilm formation pathway. In fact, the results of the current study showed that *S. aureus* isolates have possibly changed their common PIA-dependent pathway for biofilm formation and are using other pathways (including surface protein-dependent pathways). 

## Conclusion

According to the results of this study, *clf* gene possibly has an equivalent role in both groups and there is no significant difference for the role of this gene in MRSA and MSSA groups. Moreover, the results of this study suggest that *S. aureus* isolates change their common pathway for biofilm formation by acquiring methicillin resistance and the use of PIA-dependent biofilm formation pathways. This also requires further investigation. 
